# Lamivudine monotherapy as a holding regimen for HIV-positive children

**DOI:** 10.1371/journal.pone.0205455

**Published:** 2018-10-11

**Authors:** Gabriela Patten, Jonathan Bernheimer, Lee Fairlie, Helena Rabie, Shobna Sawry, Karl Technau, Brian Eley, Mary-Ann Davies, for IeDEA Southern Africa

**Affiliations:** 1 Centre for Infectious Disease Epidemiology & Research, University of Cape Town, Cape Town, South Africa; 2 Médecins Sans Frontières Khayelitsha, Cape Town, South Africa; 3 Wits Reproductive Health and HIV Institute, University of the Witwatersrand, School of Clinical Medicine, Johannesburg, South Africa; 4 Tygerberg Academic Hospital, University of Stellenbosch, Stellenbosch, South Africa; 5 Empilweni Services and Research Unit, University of the Witwatersrand, Department of Paediatrics and Child Health, Rahima Moosa Mother and Child Hospital, Johannesburg, South Africa; 6 Red Cross War Memorial Children’s Hospital, and the Department of Paediatrics and Child Health, University of Cape Town, Cape Town, South Africa; Medical University of Warsaw, POLAND

## Abstract

**Background:**

In resource-limited settings holding regimens, such as lamivudine monotherapy (LM), are used to manage HIV-positive children failing combination antiretroviral therapy (cART) to mitigate the risk of drug resistance developing, whilst adherence barriers are addressed or when access to second- or third-line regimens is restricted. We aimed to investigate characteristics of children placed on LM and their outcomes.

**Methods:**

We describe the characteristics of children (age <16 years at cART start) from 5 IeDEA-SA cohorts with a record of LM during their treatment history. Among those on LM for >90 days we describe their immunologic outcomes on LM and their immunologic and virologic outcomes after resuming cART.

**Findings:**

We included 228 children in our study. At LM start their median age was 12.0 years (IQR 7.3–14.6), duration on cART was 3.6 years (IQR 2.0–5.9) and median CD4 count was 605.5 cells/μL (IQR 427–901). Whilst 110 (48%) had no prior protease inhibitor (PI)-exposure, of the 69 with recorded PI-exposure, 9 (13%) patients had documented resistance to all PIs. After 6 months on LM, 70% (94/135) experienced a drop in CD4, with a predicted average CD4 decline of 46.5 cells/μL (95% CI 37.7–55.4). Whilst on LM, 46% experienced a drop in CD4 to <500 cells/μL, 18 (8%) experienced WHO stage 3 or 4 events, and 3 children died. On resumption of cART the average gain in CD4 was 15.65 cells/uL per month and 66.6% (95% CI 59.3–73.7) achieved viral suppression (viral load <1000) at 6 months after resuming cART.

**Interpretation:**

Most patients experienced immune decline on LM. Its use should be avoided in those with low CD4 counts, but restricted use may be necessary when treatment options are limited. Managing children with virologic failure will continue to be challenging until more treatment options and better adherence strategies are available.

## Introduction

Treatment failure among HIV-positive children is a growing concern.[1] In resource-limited settings, where access to second- or third-line combination antiretroviral therapy (cART) for HIV-positive children is frequently restricted, managing virologic failure is especially challenging.[2] Children and adolescents face a number of adherence barriers, and drug formulations are often unpalatable with large pill burdens^.^[3–5] Switching children with virologic failure, who have suspected or proven poor adherence, to a new regimen runs the risk of increasing resistant mutations, thus limiting future treatment options. The South African national guidelines recommend holding strategies for those with ongoing adherence challenges.[6]

Lamivudine monotherapy (LM) is one such holding regimen. This commonly used holding strategy reduces the pill burden to patients and allows time for adherence barriers to be addressed. It is also used whilst awaiting access to second- or third-line regimens, not always readily available. Lamivudine has a low genetic barrier to resistance and most patients with virologic failure develop the M184V mutation.[7] LM retains this HIV variant which has reduced viral fitness and may therefore slow immune decline whilst ensuring new drug resistance does not develop.[8–13]

There are few studies of the use and effectiveness of LM in children, despite it being recommended in national guidelines. Three small South African studies found that CD4 count declined slowly on LM and those with higher CD4s did not experience significant declines.[14–17] We aimed to describe the characteristics and outcomes of children placed on LM, to provide further evidence to guide the appropriate use of this treatment strategy, using data from the International epidemiologic Database to Evaluate AIDS (IeDEA) Southern Africa collaboration, which combines data across multiple sites across Southern Africa.

## Materials and methods

### Study design, setting and population

Data were collected prospectively from five IeDEA-SA cohorts in South Africa. IeDEA is a multi-regional HIV cohort collaboration, which has been previously described.[18] The requirement for informed consent has been waived as only anonymized data already collected as part of routine monitoring is contributed to the collaborative dataset. All sites have local institutional ethics approval to contribute data to IeDEA-SA analyses as follows: University of Cape Town (Khayelitsha ART Programme, Red Cross Children’s Hospital); University of Witwatersrand (Harriet Shezi Clinic, Rahima Moosa Mother and Child Hospital); and University of Stellenbosch (Tygerberg Academic Hospital).

We included data from children (<16 years) initiating cART after 2004, with a record of being on LM at some point in their treatment history. Outcomes and more detailed analysis was performed on patients who received LM for more than 90 days. Database closure ranged from October 2015 to February 2016 across sites.

### Treatment regimens

All sites made use of routine viral load (VL) monitoring and are part of the South African national ART program. The following treatment guidelines were in place during the study period: Before 2007 the recommended first-line regimen was stavudine, lamivudine and either efavirenz, or, for those starting treatment younger than three years of age, lopinavir/ritonavir (LPV/r). For those with tuberculosis or starting treatment under the age of six months, LPV/r was replaced with ritonavir alone.[19] After 2007, LPV/r was recommended for all children under the age of three years, with ritonavir super-boosting when co-treating for tuberculosis.[20, 21] From 2010, abacavir replaced stavudine in the recommended first-line regimen.

The recommended second-line regimen prior to 2010 was to change at least one of the nucleoside reverse transcriptase inhibitors (NRTIs) and replace efavirenz with LPV/r (if on efavirenz-based first-line), or switch to non-nucleoside reverse transcriptase inhibitor (NNRTI)-based second-line for those on protease-inhibitor (PI) based first-line.[19] The recommendation of switching to NNRTI-based second-line was replaced in the 2010 guidelines: for children failing LPV/r-based regimens specialist referral was recommended. For those who have failed PI-based cART, access to third-line or salvage regimens is through a national committee who reviews each patient’s treatment history individually. These regimens typically include drugs that may be difficult to access, such as new generation PIs like darunavir, or integrase inhibitors like raltegravir. Resistance testing was not routinely available during the study period.

### Key variables

Demographic, laboratory and clinical data at cART start and at the start of LM included age, gender, CD4 count and CD4%, age-adjusted weight and height z-scores, WHO stage, immune status according to WHO 2006 criteria for immunosuppression,[22] and first-line cART regimen. We considered additional characteristics at the start of LM, specifically nadir CD4% and VL prior to LM start, history of treatment interruption (defined as discontinuing all antiretrovirals for at least 7 days), and resistance profile where available. We also examined clinical data of patients whilst on LM including death, clinical disease and WHO stage 3 or 4 events. We considered disease events to be any opportunistic infection or incident HIV-associated diagnoses recorded in our dataset.

### Analysis

In this analysis, we described the characteristics of patients receiving LM summarizing continuous and categorical variables using medians and interquartile ranges, and proportions respectively. For patients with multiple episodes of LM, the first episode of at least 90 days was used. To better understand immunologic outcomes we explored mean changes in CD4 count whilst on LM, as well as immune decline, which we defined as either a drop in CD4 below 500 cells/μL for those starting LM with CD4 above 500 cells/μL, or for those starting LM with CD4 count of 500 or below, a drop in CD4 of more than 10%. We used Kaplan-Meier estimates for children on LM for at least 90 days and with at least 6 months of follow-up on LM to predict probability of immune decline whilst on LM. Survival was calculated from the date of LM start until either the date that immune decline was recorded, death, or censoring at the last clinical visit recorded before database closure. Predictors of immune decline whilst on LM were determined using Cox-proportional hazards models adjusting for age at cART start, characteristics prior to LM start including treatment interruption, nadir CD4 count and type of cART regimen, as well as duration on LM. To account for the non-independence of repeated CD4 measures taken for each patient, we used a linear mixed-effects regression models to determine the mean change in absolute CD4 count at 6 months on LM for patients with different characteristics at LM start. We also explored the immunologic and virologic outcomes for patients who resumed cART and had at least one subsequent CD4 count or VL. Kaplan-Meier estimates were used to predict the probability of virologic suppression (VL<1000) for those who resumed cART after LM. We used Cox-proportional hazards models to determine predictors of virologic suppression after cART resumption adjusting for age at LM start and current regimen after resuming cART. To explore the immunologic changes after cART resumption we calculated the CD4 slope for each child, as the difference between the CD4 at resumption of cART (up to 90 days prior and 7 days post cART resumption) and the first CD4 count taken within a year of cART resumption, divided by the time in months between these two measurements. We used linear regression to explore associations with change in CD4 after cART resumption adjusting for CD4 at cART resumption (> = 500 or <500), age at cART resumption and exposure to PI-based cART prior to LM. For multivariate models we included a number of variables relating to characteristics at cART start (age, immune suppression), at LM start (time on cART, time with virologic failure, most recent VL, most recent CD4 count, recorded PI resistance, history of treatment interruption, cART regimen, nadir CD4 count) and whilst on LM (duration of LM, experiencing an OI) *a priori*. For models relating to characteristics after cART resumption after LM we included age at LM start, current regimen after resuming cART, duration on LM, treatment interruption prior to LM, nadir CD4, CD4 at cART resumption, age at cART resumption, exposure to PI-based cART prior to LM. Final models were built by sequentially excluding variables through backwards selection using p-values. Statistical analysis was performed using Stata version 15 (STATA Corporation, College Station, TX).

## Results

### Characteristics of patients at ART and LM start

In total, 228 patients (132, 58% male) with a history of being on LM were included in the study. At cART start, median age was 7.4 years (IQR 3.2–10.0), median CD4 count was 348 cells/μL (IQR 190–616), median CD4% was 12.7% (IQR 7.6–18.0), and 118 (52%) were immune-suppressed. Most patients were initiated on efavirenz-based first-line regimens (70%), with 57 (25%) on PI-based first-line (Table 1).

**Table 1:** Characteristics of children at cART start and at start of lamivudine monotherapy (LM) (n = 228).

		Median (IQR) or N(%)	
		At cART start	At LM start
Age in years		7.4 (3.2, 10.0)	12 (7.3, 14.6)
CD4 count, n		348 (190, 616), 163	605.5 (427, 901), 218
CD4%		12.7 (7.6, 18.0), 160	22.2 (16.2, 28.2), 217
Immunosuppression^1^		118 (72%), 165	42 (20%), 207
Weight-for-age Z-score		-1.8 (-2.7, -0.9), 136	-0.8 (-1.8, -0.4), 217
Height-for-age Z-score		-2.4 (-3.3, -1.5), 163	-1.9 (-2.7, -1.0), 215
cART regimen	NNRTI-based firstline	165 (72%)	110 (48%)
	NNRTI-based secondline		13 (6%)
	PI-based firstline	57 (25%)	41 (18%)
	PI-based secondline		52 (23%)
	Unknown	3 (1%)	21 (9%)
	Non-cART	2 (1%)	
	Other non-standard	1 (0%)	2 (1%)
	Other holding regimen		2 (1%)
	Thirdline		1 (0%)
WHO stage at cART start	Stage 3 or 4	122 (54%)	
	Unknown	85 (37%)	
Year of cART Start	2002–2005	51 (22%)	
	2006–2009	110 (48%)	
	2010–2014	67 (29%)	
Duration (years) on cART at LM start			3.6 (2.0, 5.9)
CD4<500 at LM start			73 (33%)
Nadir CD4 at LM start, n			330 (176, 515), 218
Log_10_ Viral Load at LM start, n			4.2 (3.7, 4.7), 217
Time with unsuppressed VL prior to LM (>400)			421.5 (234.5, 794), 217
Treatment interruption prior to LM			21 (9%)

^1^ According to WHO 2006 criteria for immunosuppression

At LM start the median age was 12.0 years (IQR 7.3–14.6) and patients had been on cART for a median of 3.6 years (IQR 2.0–5.9), with median CD4 and CD4% of 605.5 cells/μL (IQR 427–901) and 22.2% (16.2–28.2), respectively; median VL was 4.2 log_10_ (IQR 3.7–4.7). Median time with unsuppressed VL, calculated as the total time between the first consecutive unsuppressed VL measurement and the date of LM start, was 421.5 days (IQR 234.5–794), with a median of 4 (IQR 2–5) unsuppressed VL measurements. Time with unsuppressed VL prior to LM has decreased in more recent years: among those with at least 2 years of follow-up following their first consecutive unsuppressed VL and those starting LM within 2 years of their first consecutive unsuppressed VL, the median time with unsuppressed VL declined (2002–2005: 371 days (IQR 259–519), 2006–2009: 285 days (IQR 151–472), 2010–2014: 280 days (IQR 201–425)). Prior to LM start, 110 (48%) were on efavirenz-based first-line regimens, whilst 108 (47%) had had prior exposure to PI-based regimens, one patient was on a third-line regimen, two had been on other holding regimens, and seven had unknown treatment history.

Prior to initiating LM, 116 (51%) had undergone resistance testing. Among those tested, 107 (92%) patients had resistance to lamivudine with the majority having high level resistance (96%, n = 103). Over half of patients had no PI resistance (72, 62%), whilst 10 (9%) were resistant to all PI’s.

Of 69 patients with recorded PI-exposure prior to LM and a resistance test, 38 (55%) had no PI resistance, 10 (15%) were resistant to only nelfinavir, 12 (17%) were resistant to some PIs and 9 (13%) were resistant to all PIs.

### Outcomes on LM

At study closure the median duration on LM was 309 days (IQR 94.5–644), and 174 (76%) children were on LM for more than 90 days. Four patients had been on LM on multiple occasions between periods of receiving cART.

Three patients died whilst on LM, although details on cause of death were not available. All had a CD4 at LM start >500 cells/μL and had previously been on PI-based cART (2 after first-line PI, 1 after second-line PI). Time on LM for the 3 patients ranged from less than 6 months to over 18 months, one patient dropped to a CD4<50 whilst on LM. In total, there were 114 new disease events among 53 patients whilst on LM. The median time to first disease event was 94.5 days (IQR 45–359). There were 18 (8%) patients who experienced 26 WHO stage 3 or 4 event whilst on LM, the most common being extra pulmonary tuberculosis (5), recurrent bacterial pneumonia (4), oral candidiasis (3), chronic HIV-associated lung disease (3), and acute necrotising ulcerative stomatitis, gingivitis or periodontitis (3).

At study closure, out of all 228 children, 167 patients had been switched from LM, 149 of whom resumed cART. The majority, 135, were switched to or resumed PI-based regimens (84 switched, 47 resumed, and 4 had unknown treatment history), and 12 switched to or resumed NNRTI-based regimens (7 resumed first-line, 1 resumed second-line, 3 were switched to second-line, 1 unknown). One patient was switched to a third-line regimen, after having been on PI-based second-line prior to LM, and one patient was switched to a non-standard cART regimen. Among the 18 patients not resuming cART, 4 were switched to other holding regimens (all containing 2 NRTIs only), and 14 discontinued all treatment. We did not have data on the reasons for treatment discontinuation, and the decision to stop all treatment may have been planned and recommended by a clinician, or the decision of the patient.

### Immune decline on LM

In total 147 patients were on LM for more than 90 days, had a recorded CD4 count at LM start, and at least one subsequent CD4 count measured whilst receiving LM. The median time from the CD4 count at LM start to the last CD4 on LM was 413 days (IQR 231–752). On average CD4 count measurements were taken every 132 days or every 4.3 months whilst on LM, and the median time between CD4 measurements on LM was 112 days (IQR 91–158). The median CD4 decline, from CD4 at LM start to the last recorded CD4 count on LM, was 148 cells/μL (IQR 28–414), 115 (78%) experienced a drop in CD4 ≥10%, and among the 112 with CD4 ≥500 cells/μL at LM start 46% experienced a drop in CD4 to <500 cells/μL whilst on LM.

Results from our mixed effects model predicted an average CD4 decline at 6 months on LM of 46.5 cells/μL (95% CI 37.7–55.4) from a mean CD4 of 708.4 to a mean of 661.9 cells/μL (95% CI 601.8–721.9). CD4 count at LM start influenced immune decline on LM. The predicted average decline in CD4 at 6 months on LM for those with CD4 <500 was 21.5 cells/μL (95% CI 9.7–33.2) to a mean CD4 of 306.8 cells/μL (276.2–337.5). For those with CD4 ≥500 at LM start, the predicted decline at 6 months was 55.5 cells/μL (95%CI 47.4–63.6), to a mean CD4 of 752.3 cells/μL (95% CI 704.4–800.2). To illustrate the difference in CD4 decline for patients of different characteristics, we estimated the average CD4 count at 6 months on LM for patient of varying age, duration on cART, nadir CD4 and CD4 at LM start. These results are summarized in Table 2.

**Table 2:** Estimated CD4 count decline (95% confidence intervals) at 6 months on lamivudine monotherapy (LM) for children with specific age, duration on cART, nadir CD4 and CD4 count at LM start (n = 135)*.

Age at LM start (years)	Duration on cART prior to LM (years)	Nadir CD4	CD4 at LM start = 1000	CD4 at LM start = 500
10	2	100	581.64 (403.99, 759.29)	329.11 (207.84, 450.39)
		500	489.05 (326.04, 652.06)	236.53 (107.91, 365.14)
	6	100	497.29 (330.69, 663.90)	244.77 (129.58, 359.95)
		500	404.71 (249.26, 560.16)	152.18 (23.75, 280.62)
15	2	100	652.59 (437.81, 867.36)	400.06 (233.17, 566.95)
		500	560.00 (354.73, 765.28)	307.48 (132.31, 482.64)
	6	100	568.24 (369.97, 766.52)	315.72 (162.76, 468.68)
		500	475.66 (284.04, 667.28)	223.14 (56.94, 389.34)

*This table provides the estimates for the decline in CD4 count after 6 months on LM for children of specific characteristics. Eg A child aged 10 years at the start of LM, on cART for 2 years prior to LM with a nadir CD4 of 100 and a CD4 at LM start of 1000 will have experienced a drop in CD4 count of 581.6 cells/uL after 6 months on LM

According to our definition, 76 (52%) patients experienced immune decline on LM, with an estimated probability of immune decline after 6 months on LM of 22.2% (95% CI 16.6%-29.3%). Predictors of immune decline are summarized in Table 3. Older age was associated with increased risk of immune decline; those over 9 years old had a four-fold increase in risk compare to those under 2 years (aOR 4.2, 95% CI 2.2–8.3). Those on third-line or NNRTI-based second-line were also at increased risk of immune decline compared to those on NNRTI-based first-line prior to LM start (aOR 3.43, 95% CI 1.6–7.6). Treatment interruption prior to LM, and lower nadir CD4 count were also associated with immune decline as was shorter time on LM, likely because of switch from LM in those with immune decline or clinical deterioration.

**Table 3:** Univariate and multivariable associations with a decline in CD4 of more than 10% from lamivudine monotherapy (LM) start OR drop in CD4 count below 500 in those with CD4>500 at LM start, stratified by site.

Characteristic at ART or LM start		Unadjusted HR (95% CI), n = 157	p	Adjusted HR (95% CI), n = 157	p
Age in years at ART start	<2	1	<0.001*	1.00	0.0007*
	2–6	2.24 (1.29, 3.91)		2.27 (1.16, 4.45)	
	6–9	3.08 (1.78, 5.35)		2.78 (1.34, 5.77)	
	>9	4.92 (2.95, 8.21)		4.15 (2.17, 8.34)	
Complete treatment interruption prior to LM		1.39 (0.92, 2.10)	0.12	3.69 (1.78, 7.69)	<0.001
Nadir CD4 prior to LM start	> = 500	1	<0.001*	1.00	0.0054*
	> = 350 - <500	2.07 (1.29, 3.35)		1.95 (1.14, 3.34)	
	> = 200 - <350	2.42 (1.54, 3.80)		2.30 (1.36, 3.90)	
	<200	3.45 (2.19, 5.42)		2.40 (1.42, 4.07)	
Time on LM	90 days to 6 months	1	<0.001*	1.00	0.0041*
	6 months—1 year	0.61 (0.28, 1.33)		0.53 (0.23, 1.24)	
	1–2 years	0.46 (0.21, 1.00)		0.47 (0.20, 1.08)	
	> 2 years	0.18 (0.08, 0.41)		0.23 (0.09, 0.56)	
Regimen Prior to LM start	NNRTI-based first-line	1	-	1.00	
	PI-based first or second-line	0.74 (0.52, 1.04)	0.08	1.24 (0.83, 1.87)	0.294
	Third line or NNRTI-based second-line	0.79 (0.43, 1.46)	0.46	3.43 (1.55, 7.60)	0.002
	Other / Unknown	1.23 (0.65, 2.34)	0.53	2.37 (1.13, 4.96)	0.022

*Derived from Wald's test

### Outcomes after resuming cART

Table 4 summarizes the characteristics of patients resuming cART after LM among those on LM for at least 90 days, at three time points: at LM start, on resumption of cART and 6 months thereafter. Median CD4 count returned to pre-LM levels and VL was reduced. Results from our regression analysis estimate a median CD4 slope of 15.65 cells/uL per month (IQR 1.52, 45.54). Given the small sample size we failed to demonstrate statistically significant associations with change in CD4, but those with CD4 ≥500 cells/μl on resumption of cART tended to have smaller increases in CD4 post cART resumption compared to those with CD4 <500 cells/μl (mean change 15.53 vs 36.02 per month).

**Table 4:** Characteristics of patients who resumed cART after >90 days on lamivudine monotherapy (LM), n = 103.

	LM start	cART resumption	6 months after cART resumption
Age in years*	12.01 (8.19, 14.08)	13.55 (9.60, 15.51)	14.01 (10.06, 15.81), n = 88
< = 5	14 (14%)	4 (4%)	4 (5%)
>5 - < = 10	20 (19%)	24 (23%)	18 (20%)
>10 - < = 15	50 (49%)	46 (45%)	37 (42%)
>15	19 (18%)	29 (28%)	29 (33%)
CD4 count (cells/μl)*	547 (368.5, 827.5), n = 88	357.5 (270, 575), n = 64	552 (310, 796), n = 74
<200	2 (2%)	10 (16%)	7 (9%)
> = 200–350	14 (16%)	20 (31%)	15 (20%)
> = 350–500	22 (25%)	17 (27%)	10 (14%)
> = 500	50 (57%)	17 (27%)	42 (57%)
log_10_ VL (copies/ml)*	4.18 (3.70, 4.62), n = 90	4.55 (4.01, 5.16), n = 30	2.81 (1.88, 4.36), n = 73
VL<1000	6 (7%)	3 (10%)	40 (55%)
Regimen after LM			
First-line NNRTI	52 (50%)	7 (7%)	6 (7%)
Second-line NNRTI	4 (4%)	4 (4%)	4 (5%)
First- or second-line PI	42 (41%)	89 (86%)	69 (78%)
Third-line	0 (0%)	2 (2%)	4 (5%)
Other cART	1 (1%)	1 (1%)	1 (1%)
Non-cART	1 (1%)	0 (0%)	3 (3%)
Unknown	3 (3%)	0 (0%)	0 (0%)
Discontinued all cART	0 (0%)	0 (0%)	1 (1%)

*Median (IQR), n = 103 unless otherwise indicated

Among the 103 patients resuming cART after at least 90 days on LM, 62% (64) achieved virologic suppression after resuming cART, with estimated probability of VL suppression by 6 months after cART resumption of 66.6% (95% CI 59.3–73.7). In adjusted analysis, those on PI-based cART were more likely to suppress compared to those resuming NNRTI-based cART (aOR 1.9, 95% CI 1.3–2.8). Age was associated with VL suppression, with those 5 years or younger (aOR 2.1 (95% CI 1.6–2.7), those 5 to 10 years of age (aOR 1.9 (95% CI 1.5–2.3) and those aged 10 to 13 years (aOR 1.1 (95% CI 0.8–1.3) more likely to suppress compare to those older than 13 years. Regimen prior to LM was not associated with virologic suppression after resumption of cART and was not included in the final model.

Among patients on PI-based regimens prior to LM, on resumption of PI-based cART 21/36 (58%) achieved VL suppression. Among the 46 patients on NNRTI-based first-line prior to LM, a third (2/6) patients suppressed on NNRTI-based regimens after resuming cART, while 31/40 (78%) suppressed on PI-based cART.

## Discussion

The majority of children placed on LM experienced declines in CD4, 8% experienced WHO stage 3 or 4 events, and 3 children died. Those with higher CD4 count at LM start, lower nadir CD4 and older age experienced bigger absolute declines in CD4 count. Our composite outcome of immune decline (either a drop in CD4 below 500 cells/μL for those starting LM with CD4 above 500 cells/μL, or for those starting LM with CD4 count of 500 or below, a drop in CD4 of more than 10%) was experienced by roughly half of patients. Predictors of immune decline were lower nadir CD4 count, and having a history of treatment interruption. After resuming cART, the majority of patients achieved virologic suppression and some immune recovery. Adolescents and those on NNRTI-based cART were less likely to achieve virologic suppression on resumption of cART.

Most children placed on LM had failed to suppress virologically for over a year. Time with unsuppressed VL prior to LM has decreased in more recent years suggesting a trend to earlier adoption of this strategy. About half our population had limited treatment options and were placed on LM after exposure to PI-based regimens, with 10 children having documented resistance to all PIs. The other half of children placed on LM had only had exposure to NNRTI-based first-line regimens and were placed on this strategy instead of switching directly to PI-based second-line. After LM few of these children resumed NNRTI-based cART and very few managed to suppress on this regimen. The majority resumed PI-based regimens and had good virologic outcomes. For these children being on LM delayed the initiation of second-line whilst risking further immune decline, although it is possible that barriers to adherence were addressed during this time so that adherence was optimized by the time second-line was started.

This study represents the largest cohort of children on LM, providing the most evidence on the use of this strategy. We explored outcomes of patients on resumption of cART following LM; only one US-based study of 36 patients has reported on similar outcomes.[23] Like all previous reports on LM outcomes, our observational study on immune decline on LM is subject to confounding by indication. Those with clinical or immunological deterioration on LM may be more likely to resume cART sooner, whilst those who are awaiting third-line and have no option but to remain on LM, may experience larger CD4 declines and longer duration on LM. This is shown in our results which indicate that those with shorter duration on LM and those on third-line or requiring salvage regimens were at higher risk of immune decline.

We had no data on adherence, and therefore could not directly assess the effect of LM as a strategy to improve adherence, or determine how much adherence influenced immunologic outcomes on LM or outcomes on resumption of cART. We did not have data on the reason for starting the patient on LM, and could only infer from the patient’s treatment history. Resistance testing was not available for all patients. VL monitoring is not routinely performed for those on non-supressive regimens like LM, so we could not assess the virologic consequences of this strategy.

Our study had a larger and older study population than those previously published, but our results are largely in agreement. Linder et al reported generally larger immune declines, but children in their study started LM with lower CD4 counts, and had longer durations on LM (mean duration of over 2 years compared to our median of under 1 year).[15] A similar proportion of patients experienced stage 3 or 4 clinical events, but in our study 3 patients died whilst on LM, although we had no details on the cause. No other study has reported deaths while on this strategy. Our results are not representative of children <3 years of age, and it is perhaps advisable to avoid LM in young children given the lack of data in this age group.

There has been some attempt to prospectively compare outcomes of holding regimens like LM, with other strategies to manage virologic failure in children. The IMPAACT P1094 study enrolled children failing non-NNRTI-based cART, and aimed to compare outcomes of those randomized to continue with failing cART versus changing to a holding regimen of lamivudine or emtricitabine monotherapy. The study was discontinued due to lack of enrolment, but results from the small study population indicate greater immune decline for those on holding regimens.[24] Fairlie et al report on a US-based cohort of children comparing strategies for managing children with virologic failure, including holding regimens such as LM, but were unable to reach conclusions about holding regimens as compared to switching to a new regimen, continuing on a failing regimen or discontinuing all cART, due to sample size constraints.[25] An observational study of children from a US-based cohort who interrupted treatment, either by discontinuing all cART or by being placed on LM, found better immune rebound after restarting cART among those on LM compared to restart after complete treatment interruption.[23]

This study provides useful evidence on the outcomes of children on LM and after resuming cART, providing guidance on which patients are at greater risk of immune deterioration on LM. This strategy should be avoided among children with low CD4 counts, and patients on LM require careful monitoring. Half of our study population were failing PI-based regimens and had limited treatment options available to them. As more HIV-positive children are started on PI-based cART due to earlier HIV diagnosis, it is likely that an increasing number of children will experience virological failure on PI-based cART. As long as treatment options for children remain scarce, and the risk of poor adherence and resistance continues, the use of holding regimens like LM, could increase as the numbers of children failing treatment grow. There is an urgent need for evidence to inform the optimal management of children with virological failure, as well as to inform second- and third-line options for children in resource-limited settings.[26] As new antiretrovirals such as the integrase inhibitors dolutegravir and raltegravir become more widely accessible, giving children more treatment options, the use if LM as an initial strategy to manage virologic failure in children should be reconsidered. Our findings that adolescents were less likely to achieve virologic suppression on resumption of cART, highlights the challenge of managing treatment failure and maintaining life-long adherence in this vulnerable population. Effective adherence support strategies are urgently needed as the number of adolescents on cART continues to grow.

To understand how best to manage virologic failure in children and adolescents, comparison between switching patients directly to second-line, keeping them on their failing regimen or discontinuing all treatment is needed. This will allow a better understanding of the benefits and potential risks of using holding regimens like LM.

## Conclusion

In conclusion, most patients experienced immune decline on LM, and its use should be avoided in those with low CD4 count. LM may be a useful strategy in the management of patients with virologic failure and poor adherence when treatment options are limited, but close clinical and immunological monitoring is required. Managing children with virologic failure will continue to be challenging until more treatment options and better adherence strategies are available.

## References

[1] Davies MA, Moultrie H, Eley B, Rabie H, Van Cutsem G, Giddy J, et al Virologic failure and second-line antiretroviral therapy in children in South Africa—the IeDEA Southern Africa collaboration. J Acquir Immune Defic Syndr. 2011;56(3):270–8. 10.1097/QAI.0b013e3182060610 21107266 PMC3104241

[2] Meyers T, Moultrie H, Naidoo K, Cotton M, Eley B, Sherman G. Challenges to pediatric HIV care and treatment in South Africa. J Infect Dis. 2007;196 Suppl 3:S474–81. 10.1086/521116 18181697

[3] Steele RG, Nelson TD, Cole BP. Psychosocial functioning of children with AIDS and HIV infection: review of the literature from a socioecological framework. J Dev Behav Pediatr. 2007;28(1):58–69. 10.1097/DBP.0b013e31803084c6 17353739

[4] Agwu AL, Fairlie L. Antiretroviral treatment, management challenges and outcomes in perinatally HIV-infected adolescents. J Int AIDS Soc. 2013;16:18579 10.7448/IAS.16.1.18579 23782477 PMC3687074

[5] Haberer J, Mellins C. Pediatric adherence to HIV antiretroviral therapy. Curr HIV/AIDS Rep. 2009;6(4):194–200. 19849962 10.1007/s11904-009-0026-8 PMC2967363

[6] National Consolidatd Guidelines for the prevention of mother-to-child transmission of HIV (PMTCT) and the management of HIV in children, adolescents and adults. National Department of Health, South Africa, 2015.

[7] Deval J, White KL, Miller MD, Parkin NT, Courcambeck J, Halfon P, et al Mechanistic basis for reduced viral and enzymatic fitness of HIV-1 reverse transcriptase containing both K65R and M184V mutations. J Biol Chem. 2004;279(1):509–16. 10.1074/jbc.M308806200 14551187

[8] Opravil M, Klimkait T, Louvel S, Wolf E, Battegay M, Fux CA, et al Prior therapy influences the efficacy of lamivudine monotherapy in patients with lamivudine-resistant HIV-1 infection. J Acquir Immune Defic Syndr. 2010;54(1):51–8. 10.1097/QAI.0b013e3181bef889 19838125

[9] Gianotti N, Tiberi S, Menzo S, Danise A, Boeri E, Galli L, et al HIV-1 replication capacity and genotype changes in patients undergoing treatment interruption or lamivudine monotherapy. Journal Med Virol. 2008;80(2):201–8. 10.1002/jmv.21085 18098142

[10] Quan Y, Brenner BG, Oliveira M, Wainberg MA. Lamivudine can exert a modest antiviral effect against human immunodeficiency virus type 1 containing the M184V mutation. Antimicrob Agents Chemother. 2003;47(2):747–54. 10.1128/AAC.47.2.747-754.2003 12543687 PMC151747

[11] Castagna A, Danise A, Menzo S, Galli L, Gianotti N, Carini E, et al Lamivudine monotherapy in HIV-1-infected patients harbouring a lamivudine-resistant virus: a randomized pilot study (E-184V study). AIDS. 2006;20(6):795–803. 10.1097/01.aids.0000218542.08845.b2 16549962

[12] Devereux HL, Emery VC, Johnson MA, Loveday C. Replicative fitness in vivo of HIV-1 variants with multiple drug resistance-associated mutations. J Med Virol. 2001;65(2):218–24. 11536226 10.1002/jmv.2023

[13] Soria A, Danise A, Galli L, Tiberi S, Seminari E, Cossarini F, et al Viro-immunological dynamics in HIV-1-infected subjects receiving once-a-week emtricitabine to delay treatment change after failure: a pilot randomised trial. J Clin Virol. 2010;47(3):253–7. 10.1016/j.jcv.2009.12.007 20056480

[14] Lazarus EM OK, Fairlie L, Untiedt S, Violari A, Laher F, Evans D, Levin L. Lamivudine Monotherapy as a Holding Strategy in HIV-Infected Children in South Africa. J AIDS Clin Res. 4(10).

[15] Linder V, Goldswain C, Adler H, Carty C, Harper K, Jackson V, et al Lamivudine Monotherapy: Experience of Medium-term Outcomes in HIV-infected Children Unable to Adhere to Triple Therapy. Pediatr Infect Dis J. 2016;35(7):e199–205. 10.1097/INF.0000000000001156 27031256

[16] Linder V, Goldswain C, Boon G, Carty C, Jackson V, Harper K, et al Lamivudine monotherapy as a safe option for HIV-infected paediatric clients with adherence challenges: new evidence from a large South African cohort. J Int AIDS Soc. 2014;17(4 Suppl 3):19763 10.7448/ias.17.4.19763 25397507 PMC4225417

[17] Rabie H, Essack G, Cotton M, editors. Monotherapy with lamivudine in HIV-infected children: the experience at Tygerberg hospital. South African HIV Clinicians Society Conference; 2012 25–28 November 2012; Cape Town, South Africa.

[18] Egger M, Ekouevi DK, Williams C, Lyamuya RE, Mukumbi H, Braitstein P, et al Cohort Profile: the international epidemiological databases to evaluate AIDS (IeDEA) in sub-Saharan Africa. Int J Epidemiol. 2012;41(5):1256–64. 10.1093/ije/dyr080 21593078 PMC3465765

[19] National Antiretroviral Treatment Guidelines. In: National Department of Health SA, editor.: Jacana; 2004.

[20] Ren Y, Nuttall JJ, Egbers C, Eley BS, Meyers TM, Smith PJ, et al Effect of rifampicin on lopinavir pharmacokinetics in HIV-infected children with tuberculosis. J Acquir Immune Defic Syndr. 2008;47(5):566–9. 10.1097/QAI.0b013e3181642257 18197120

[21] Chadwick EG, Capparelli EV, Yogev R, Pinto JA, Robbins B, Rodman JH, et al Pharmacokinetics, safety and efficacy of lopinavir/ritonavir in infants less than 6 months of age: 24 week results. AIDS. 2008;22(2):249–55. 10.1097/QAD.0b013e3282f2be1d 18097227

[22] WHO. Antiretroviral therapy of HIV infection in infants and children: towards universal access. Switzerland: 2006. 23741772

[23] Rakhmanina N, Lam KS, Hern J, Young HA, Walters A, Castel AD. Interruptions of antiretroviral therapy in children and adolescents with HIV infection in clinical practice: a retrospective cohort study in the USA. J Int AIDS Soc. 2016;19(1):20936 10.7448/IAS.19.1.20936 27797320 PMC5087211

[24] Agwu A, Warshaw M, Siberry GK, Melvin A, McFarland E, Wiznia A editors. 3TC/FTC monotherapy vs. continuing failing cART as a bridging ART strategy in persistently nonadherent HIV-infected youth with M184V resistance: results of IMPAACT P1094. 6th International Workshop on HIV Pediatrics; 2014; Melbourne, Australia.

[25] Fairlie L, Karalius B, Patel K, van Dyke RB, Hazra R, Hernan MA, et al CD4+ and viral load outcomes of antiretroviral therapy switch strategies after virologic failure of combination antiretroviral therapy in perinatally HIV-infected youth in the United States. AIDS. 2015;29(16):2109–19. 10.1097/QAD.0000000000000809 26182197 PMC4612147

[26] Lazarus E, Nicol S, Frigati L, Penazzato M, Cotton MF, Centeno-Tablante E, et al Second- and Third-line Antiretroviral Therapy for Children and Adolescents: A Scoping Review. Pediatr Infect Dis J. 2017;36(5):492–9. 10.1097/INF.0000000000001481 28403052

